# Molecular characterization of *Mycobacterium bovis *isolates from Ethiopian cattle

**DOI:** 10.1186/1746-6148-6-28

**Published:** 2010-05-27

**Authors:** Demelash Biffa, Eystein Skjerve, James Oloya, Asseged Bogale, Fekadu Abebe, Ulf Dahle, Jon Bohlin, Berit Djønne

**Affiliations:** 1 Center for Epidemiology and Biostatistics, Norwegian School of Veterinary Science, P.O. Box 8146, N-0033, Oslo, Norway; 2 Department of Veterinary Public Health and Preventive Medicine, Makerere University P.O. Box, 7062, Kampala, Uganda; 3 College of Veterinary Medicine, Nursing and Allied Health Sciences, Tuskegee University, Williams-Bowie, Bldg 114, Tuskegee, AL 36088, USA; 4 Institute of General Practice and Community Medicine, Faculty of Medicine, University of Oslo, P.O. Box 1130 Blindern, Oslo, Norway; 5 Department of Bacteriology and Immunology, Division of Infectious Disease Control, Norwegian Institute of Public Health, P.O. Box 4404 Nydalen N0-0403 Oslo, Norway; 6 Department of Animal Health, National Veterinary Institute, P.O. Box 750 dep, N-0106 Oslo, Norway

## Abstract

**Background:**

Bovine Tuberculosis (BTB) is a widespread and endemic disease of cattle in Ethiopia. Information relating to genotypic characteristics of *Mycobacterium bovis *strains affecting the cattle population in Ethiopia is limited. We carried out molecular characterization of *M. bovis *strains isolated from BTB infected cattle using the spoligotyping technique. The relationship between distribution of spoligotypes and recorded variables was also investigated. A new approach that can numerically reflect the degree of genetic polymorphism in a *M. bovis *population was also developed. The study was conducted from July 2006 to January 2007 in cattle slaughtered at five representative abattoirs in Ethiopia.

**Results:**

Forty-five *M. bovis *isolates were obtained from 406 pathologic tissue specimens collected from 337 carcasses with lesions compatible with BTB. Twelve spoligotypes were identified from 34 distinct strains; with SB1176 as a dominant spoligotype (41.2% of the isolates) followed by SB0133 (14.7%). Comparison of spoligotypes with an *M. bovis *global database http://www.mbovis.org revealed six new spoligotypes which were subsequently registered in the database with international identification codes of SB1517, SB1518, SB1519, SB1520, SB1521 and SB1522. The majority of strains were obtained from cattle slaughtered at Addis Ababa abattoir. On the basis of the Spoligotype Evolutionary Index, SEI (a numeric expression approach to make standardized comparison of spoligotype evolution), *M. bovis *isolates from Ethiopia were relatively more heterogeneous (SEI = 3.2) compared to isolates from other countries. This might be attributed to extensive livestock movement linked to trading or seasonal migration, high degree of livestock mingling, and also diversities of the country's agricultural and livestock ecosystems, in addition to lack of disease control measures that led to high infection prevalence. Multiple spoligotype infection was recorded in nine (50%) of infected carcasses and this may indicate the prevailing high degree of super infection.

**Conclusions:**

This study provided molecular evidence for the widespread distribution of *M. bovis *in the cattle population in Ethiopia. It also demonstrated a relatively high degree of genetic polymorphism of the isolates. Further molecular investigation of *M. bovis *strains in humans and other domestic animals is recommended in order to elucidate the zoonotic importance as well as reservoirs and pattern of transmission among various hosts.

## Background

Ethiopia, a country whose economy is largely dependent on agriculture, possesses the largest livestock resource in Africa. Livestock sector contributes about 18% of the national GDP and 30% of the agricultural employment [1]. However, infectious diseases, accounting for 30-50% of the total annual losses [2], remain a major impediment to Ethiopia's livestock economy. Bovine tuberculosis (BTB) is an important infectious disease of Ethiopian cattle whose existence has been documented in almost all parts of the country. No recognizable control programs are implemented except the routine abattoir inspection that involves whole or partial condemnation of infected carcasses for the purpose of protecting consumers' health. Even then, routine meat inspection procedures with qualified professionals are only practised in a handful of municipal and export abattoirs throughout the country. It is estimated that more than half of slaughtered animals each year are illegally processed in backyard system without undergoing proper inspection, thus posing a great health risk to the consumers. This is further exacerbated by the low sensitivity of routine meat inspection to detect carcasses with tuberculosis lesions, and subsequently infected meat can get approved for human consumption [3].

*Mycobacterium bovis (M. bovis)*, the causative agent of BTB, is a member of the Mycobacterium Tuberculosis Complex (MTC) that also includes *M. tuberculosis, M. africanum*, *M. canetti *and *M. microti*. BTB is a worldwide animal health problem and remains a major threat to public health in countries in which people live in close proximity with their cattle and where milk is not pasteurized [4,5]. The correlation between the prevalence of *M. bovis *infection in humans and in local cattle populations highlights the potential threat of this disease to humans [5]. BTB also has a significant socio-economic impact, as it can affect international trade of animals and animal products [6].

Traditional characterization of bacterial pathogens is becoming increasingly unsuitable for modern epidemiology because it is based on variable phenotypes that are not necessarily related to genetic descent [7]. In the last decades, fast, easy and sensitive methods based on the detection of genetic diversities of *Mycobacterium *species and typing of individual strains for epidemiological analysis have been developed [8]. Molecular methods have become very tightly integrated with traditional epidemiological tracing of tuberculosis and provide a paradigm for such integration at the local and international levels [7]. Several typing techniques have been used to characterize isolates of *M. bovis*, with IS*6110 *restriction fragment length polymorphism (RFLP), spoligotyping and multilocus variable number of tandem repeat analysis (MLVA) as the most frequently used.

Spoligotyping is a PCR based technique that detects polymorphism within the genomic direct repeat (DR) locus which consists of multiple sequences interspersed with non-repetitive spacer sequences (spacers) [9]. It is well suited for epidemiological studies of *M. bovis *isolates, as it has a high level of discriminatory ability allowing disclosure of several epidemiological relationships [10]. Simplicity and reproducibility of the method has helped to establish an international *M. bovis *spoligotype database http://www.mbovis.org (designed and managed by Noel Smith and Rainer Hilscher and hosted on University of Sussex servers) that enables the recognition of new and emerging strains as well as comparison of geospatial distribution and spread of specific strains.

Even though Ethiopia is one of the countries in Africa with a high prevalence of BTB, there is limited information about the genotypic characteristics of *M. bovis *strains infecting animals and in-contact human communities. In addition to its use in designing a more targeted control measure, the availability of such information would help study phylogenetic characteristics of the organism that in turn provide new insight into the natural history of BTB [11].

The aim of this study was to use spoligotyping tool to characterise *M. bovis *strains causing BTB in Ethiopian cattle. Secondly, to perform a comparative evaluation of the degree of genetic polymorphism of DR regions in *M. bovis *populations based on a new approach of numerical expression referred to as Spoligotype Evolutionary Index (SEI).

## Methods

### Detection of tuberculosis infections by detailed necropsy examination

During July 2006 to January 2007, carcasses of 3322 cattle of different age groups, sex, breeds and classes/types (bull, cow, calf or steer) were subjected to detailed necropsy examinations for the detection of gross lesions compatible with BTB. The animals originated from six geographic regions across the country and were slaughtered at five abattoirs including four Municipal (Addis Ababa, Adama, Hawassa and Yabello) and one export (Melge-Wondo) abattoirs. For technical procedures of detailed necropsy examinations of carcasses, the readers are referred to the previously published paper from the same study [3].

### Collection of specimens

A total of 406 pathologic tissue specimens were collected aseptically from 27 different tissues/organs of 337 carcasses suspected to have been infected with TB. These included lymph nodes such as tracheobronchial, parotid, retropharyngeal, mediastinal, mesenteric, submaxilliary, precrural, prescapular, supramammary, inguinal, apical, ischiatic, iliac sternal and portal and organs/tissues such as lungs, liver, kidneys, spleen, mammary tissue, intestines, heart and inter-costal muscles, abdominal and thoracic membranes, meninges and vertebral bones. The specimens were labelled and sealed separately, and temporarily stored at -20°C in the veterinary division of the Ministry of Agriculture (MoA), or in health centres of the respective districts where the abattoirs were located. Collected specimens were brought together and kept temporarily at the laboratory facilities of the Mycobacteriology Division of the Infectious Disease Department of the Ethiopian Health and Nutrition Research Institute (EHNRI), Addis Ababa, before being transferred to a BSL-3 laboratory at the National Veterinary Institute, Oslo, Norway for the necessary mycobacteriological analysis.

### Culturing and identification of mycobacteria

Specimen processing and culturing for isolation of mycobacteria was carried out following standard procedure described by OIE [12]. Presumptive mycobacterial colonies (based on growth rate, colony characteristic and detection of acid fast bacilli by the Ziehl-Neelsen staining technique) were further subjected to Accu-Probe Mycobacterium Tuberculosis Complex (MTC) culture identification test as described in the manufacturer's manual (Gene-Probe, Inc. San Diego, California, USA). This rapid DNA probe test is based on the ability of a chemiluminescent labelled probe of single-stranded DNA to complement with ribosomal RNA of mycobacteria which form a stable DNA-RNA hybrid. Positive and negative control strains of *M. tuberculosis *(H37Rv) and *M. avium *(ATCC 25291) were obtained from the mycobacterium culture collection of the National Veterinary Institute, Oslo.

### Spoligotyping

Isolation and purification of chromosomal (template) DNA from the mycobacterial culture was done using cetyl-trimethyl-ammonium bromide (CTAB) method as described in the manual for spoligotyping (Isogen, Life Science, The Netherlands). Briefly, a loopful of mycobacterial colonies grown on Stonebrinck Media was heat-inactivated by incubation at 80°C for 20 min in 400 μl of 1× TE buffer. The cells were lysed by lysozyme, SDS and proteinase K solution. Purification was carried out by adding CTAB/NaCl solution and incubating at 65°C for 10 min. After adding 750 μl of chloroform and centrifuging for 5 min at 13,000 rpm (Mikr020, Hettich-Zentrifugen, Germany), the aqueous supernatant was transferred into fresh micro centrifuge tubes and mixed with isopropanol and centrifuged for 15 min. The precipitated DNA pellet was rinsed with 70% ethanol. The pellet was eventually dried for 30 min and redissolved in 20 μl of 1×TE buffer. The concentration and purity of extracted DNA was measured using nanodrop spectrophotometer (Thermo scientific Nanodrop™ 1000, Wilmington, Delaware, USA).

Spoligotyping of the 48 MTC isolates was carried out at the National Reference Laboratory for Mycobacteria, Division of Infectious Disease Control, Norwegian Institute of Public Health, Oslo, Norway as described in the manual (Isogen). Amplification of the spacer DNA was carried out in a Corbett thermal cycler (Biotech International) using 30 cycles of DNA denaturation for 1 min at 96°C; annealing for 1 min at 55°C and elongation for 30 sec at 72°C. The presence of amplified products was visualized by electrophoresis at 100 V for 30 min in a 2% agarose gel stained with ethidium bromide (0.5 μg/ml). The amplified spacers were then hybridized with the known oligonucleotide spacers ligated to the membrane (Isogen).

Hybridization was performed by incubating the membrane at 60°C for 1 hr. Hybridized DNA was detected by incubating the membrane in 40 ml of ECL detection liquid (Amersham, Bioscience, UK) for 1 min and visualized by exposure of a light sensitive film for 20 min (Hyper film TM ECL, Amersham Bioscience, UK). The membrane was then analyzed by recording the presence or absence of signals at the sites of the individual probes. *M. tuberculosis *(H37Rv) and *M. bovis *BCG P3, which were obtained from the National Institute of Public Health, Oslo, Norway, were included as controls.

### Data collection and analysis

Animal factors believed to be associated with BTB were recorded. These included age, sex, type (class) and breed. Cattle were classified as young (<6 years) and old (>6 years) based on dentition characteristics [13] and/or information obtained from animal owners or farm records. Geographic origin of the animals and the system under which they were managed were also recorded. Depending on characteristics and body distribution, lesions were categorized as localised or generalised. Fisher's exact test was used to investigate possible associations between distribution of specific spoligotypes and variables such as breed, geographic origin, management system and type/severity of lesions. For this purpose, isolates from same carcass showing identical spoligotypes were considered as one strain, and infected animals were assumed to originate from different farms.

Differentiation of *M. bovis *strains by spoligotyping was done based on the binary system as described by Dale *et al*. [14]. The results were entered in a computer-based strain identification system described in central database website http://www.mbovis.org. Strains with a code that had not been described previously were considered as new. The spoligotyping results were analysed using Bionumerics software program version 5 (Applied Maths, Kortrijk, Belgium). The dendrogram and strain clustering were generated by the unweighted-pair group method with Arithmetic Mean (UPGMA). The optimization and tolerance were set at 1.00%. A cluster was defined as a group of isolates with ≥ 90% genetic similarities. A dataset for individual variables and spoligotypes was established in an Excel spreadsheet (Microsoft Excel 2003). Frequency distribution of spoligotypes with respect to abattoir was presented graphically using clustered column with a 3-D visual effect chart. The association between spoligotype distribution and putative factors was investigated using Fisher's exact test (two tailed). Distribution of multiple spoligotypes with respect to abattoir, geographic origin and individual characteristics of animal is presented in tabular form.

Discriminatory power of the spoligotyping method was established by calculating Hunter-Gaston Diversity Index (HGDI) [15] using the in silico website of the university of the Basque Country http://insilico.ehu.es/mini_tools/discriminatory_power/show=formula. HGDI was based on the following numerical expression:, where s is number of distinct spoligotypes; n is number of unrelated strains (identical spoligotypes isolated from same carcass were considered as one); x_j _is frequency of i^th ^spoligotype.

### Comparison of genetic polymorphisms within the *M. bovis *population

In order to compare the genetic similarity or difference occurring among the populations (sets of isolates) of Mycobacterium Tuberculosis Complex (MTC) isolates obtained from various geographic regions across the globe, the Spoligotype Evolutionary Index (SEI) was developed and employed. The SEI is a numerical expression approach that helps to make standardized comparison of spoligotype diversities and the extent of evolution of DR regions as described by the number of spacers absent.

Where;

S = Number of distinct spoligotypes

N = Number of strains (unrelated)

sdi^th ^= No. of spacers absent in the n^th ^spoligotype

sdc = No. of spacers absent in the control strain

O = Total oligonucleotide spacers

f (i^th^) = Frequency of i^th ^spoligotype

K = Constant (proportion of spacers absent in the control strain)

Accordingly, for *M. bovis *BCG (reference strain) the number of spacers absent (sdc) = 8; O = 43 and K = 8/43 = 0.18. Then equation 1 is reduced to the following expression for comparison of genetic polymorphisms of DR region within *M. bovis *isolates obtained from different geographic regions/countries.

Interpretation of SEI is based on comparing values with the" ancestral"/reference strain (*M. bovis *BCG) whose SEI score is equal to 0.18 (the lowest value a spoligotype can have) indicating the minimum change that can happen to DR region (as reflected by presence/absence of spacers). SEI can assume any absolute number between 0.18 and ∞. Therefore, the closer the value of SEI to 0.18, the lower the polymorphism of DR region of a given sets of spoligotypes. Similarly, the higher the value of SEI, the higher the polymorphism of DR in a given set of strains (several spoligotypes with many missing spacers). This shows that even if a spoligotype with few spacers deleted (for example SB0120/*M. bovis *BCG) occurs in a high frequency, its SEI score is 0.18 suggesting that it has undergone a slight or no polymorphism in DR region. On the other hand, higher SEI score is a desirable characteristic of sets of many spoligotypes with high polymorphism in DR region.

## Results

Of 406 tissue specimens collected and cultured, 25.9% (105/406) yielded mycobacteria. Out of these, 48 isolates were found to belong to MTC by the AccuProbe method. Spoligotyping identified 45 of the MTC isolates from 18 cattle carcasses as *M. bovis *by lack of spacers 3, 9, 16 and 39-43, a unique property that distinguishes most *M. bovis *strains from other members of the group. Three isolates did not yield any results with spoligotyping (absence of hybridization signals).

Both control strains yielded standard patterns of spacer arrangements; absence of spacers 20-21 and 33-36 and presence of spacers 39-43 for *M. tuberculosis *H37Rv and absence of spacers 3, 9, 16 and 39-43 for *M. bovis *BCG P3.

**Figure 1:**
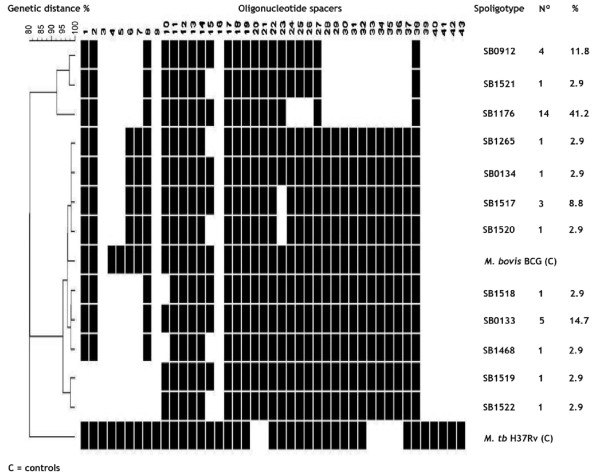
**Cluster analysis showing genetic relationship among Ethiopian *M. bovis *strains (n = 34) obtained from cattle revealing gross pathologic lesions compatible with bovine tuberculosis**. The dendrogram was built using unweighted pair group method with arithmetic average method (UPGMA). *M. bovis *BCG (SB0120) and *M. tuberculosis *(H37Rv) were included as controls. Columns on the right show international spoligotype name and frequency and the corresponding percent of isolation.

Figure 1 presents spoligotype patterns and frequency of occurrence of *M. bovis *spoligotypes in the whole material. Considering identical spoligotypes from the same animal as one, a total of 34 unrelated strains were identified. The strains were grouped into 12 spoligotypes: All lacked other spacers in addition to those characteristic of spoligotype SB0120. SB1176, the most frequently isolated spoligotype (41.2%), was characterized by absence of spacers 4-7, 24-26 and 28-37. SB0133, the second most frequently isolated (14.1%), was much closer to *M. bovis *BCG except that it lacked spacers 4-7. SB1517 (characterized by absence of spacers 4-5 and 22), and SB0912 (characterized by absence of spacers 4-7, and 28-37) accounted for 11.8% of the isolates each.

Comparison of the patterns with the global *M. bovis *database revealed six spoligotypes that had not been reported earlier. These were subsequently given an international identification code and registered in the global *M. bovis *database http://www.mbovis.org as SB1517, SB1518, SB1519, SB1520, SB1521 and SB1522. At ≥ 90% genetic relatedness, the 12 spoligotypes were grouped into two clusters (Figure 1). The first cluster included three spoligotypes namely; SB0912, SB1521 and SB1176, which were characterized by the absence of spacers 4-7 and 28-37, and contained spacers 10-14 and 17-23. The second cluster, characterized by presence of spacers 11-14 and 24-38, included nine closely related types; SB1265, SB0134, SB1517, SB1520, SB1518, SB0133, SB1468, SB1519 and SB1522.

**Figure 2:**
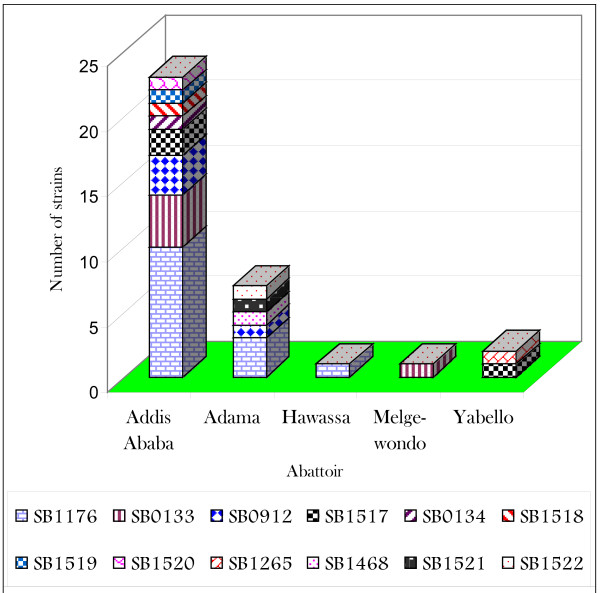
Frequency distribution of spoligotypes of Ethiopian *M. bovis *strains by abattoir

Assessment of the relationship between distribution of specific spoligotypes and breed, geographic origin, management system and severity of lesions, did not show significant statistical association (Table 1). Figure 2 presents distribution of spoligotypes by abattoir. Eight spoligotypes were recorded at Addis Ababa abattoir and this was followed by 5 spoligotypes from Adama abattoir. Among the unique spoligotypes, three (SB1518, SB1519 and SB1520) were only isolated from Addis Ababa abattoir, two (SB1521 and SB1522) from Adama Municipal abattoir and one (SB1517) from Addis Ababa and Yabello abattoirs.

**Table 1:** Distribution of *M. bovis *spoligotypes with respect to cattle breed, geographic origin of animal, prevailing management system and severity of pathologic lesions

	*M. bovis *spoligotypes													
		
Factor	Category	SB1176	SB1517	SB0133	SB1518	SB1265	SB1468	SB0134	SB0912	SB1519	SB1520	SB1521	SB1522	Total [%]
Breed	Local	8	1	3	-	1	1	-	1	-	-	1	1	17 [50.0]
	Cross	1	1	1	-	-	-	-	1	-	1	-	-	5 [14.7]
	Exotic	5	1	1	1	-	-	1	2	1	-	-	-	12 [35.3]
Origin	Southern	1	1	1	-	1	-	-	-	-	-	-	-	4 [11.8]
	Addis Ababa	5	2	3	-	-	-	1	2	-	1	-	-	14 [41.2]
	Central	8	-	1	1	-	1	-	2	1	-	1	1	16 [47.1
Management	Small scale	6	-	1	-	-	1	-	1	-	-	1	1	11 [32.4]
	Private	3	2	2	-	-	-	1	2	-	1	-	-	11 [32.4]
	Large scale	3	-	1	1	-	-	-	1	1	-	-	-	7 [20.6]
	Transhumance	2	1	1	-	1	-	-	-	-	-	-	-	5 [14.7]
Lesion severity	Localized	8	-	2	1	-	1	-	1	1	-	1	1	16 [47.1]
	Disseminated	6	3	3	-	1	-	1	3	-	1	-	-	18 [52.9]

Fisher's exact test for breed = 0.77; Origin = 0.46; Management system = 0.87; TB severity = 0.35

Nine out of 18 (50%) BTB confirmed cases were found to harbour two or more spoligotypes (Table 2). These cases were recorded from Addis Ababa, central and southern regions. SB1176 was dominant in all cases of multiple spoligotype infections. Five distinct spoligotypes were seen to occur simultaneously in two carcasses (cases 321 and 422), where four spoligotypes (SB1176, SB1517, SB0133, and SB0912) were present in both cases while SB0134 was present in 321 and SB1520 in 422. The discriminatory power (HGDI) of the spoligotyping method was calculated to be 0.81. Also on the basis of the above findings (the number of distinct spoligotypes (S) = 12, total number of unrelated strains (N) = 34, total oligonucleotide spacers for DR region (O) = 43, and the constant (K) = 0.18), the SEI of spoligotype evolution for the current population of Ethiopian *M. bovis *isolates was computed to be 3.2.

**Table 2:** Occurrence of multiple *M. bovis *spoligotypes with respect to abattoir, geographic origin and characteristics of BTB infected cattle in Ethiopia.

Case code	Abattoir	Geographic| origin	Host characteristics				
			Sex	Age	Class	Breed	Spoligotype
177	Addis Ababa	Addis Ababa	Male	Old	Bull	Local	SB1176
							SB0133
321	Addis Ababa	Addis Ababa	Female	Young	Cow	Exotic	SB1176
							SB0133
							SB1517
							SB0912
							SB0134
328	Addis Ababa	Central	Female	Old	Cow	Exotic	SB1176
							SB1518
332	Addis Ababa	Central	Male	Young	Calf	Exotic	SB1176
							SB0912
330	Addis Ababa	Central	Female	Young	Calf	Exotic	SB1519
331	Addis Ababa	Central	Female	Young	Calf	Exotic	SB1176
422	Addis Ababa	Addis Ababa	Male	Young	Bull	Exotic	SB1176
							SB0133
							SB0912
							SB1517
							SB1520
81	Addis Ababa	Addis Ababa	Male	Young	Steer	Local	SB1176
94	Addis Ababa	Addis Ababa	Female	Old	Cow	Exotic	SB 1176
114	Addis Ababa	South	Male	Old	Bull	Local	SB0133
230	Addis Ababa	Central	Male	Old	Steer	Local	SB 1176
369	Addis Ababa	South west	Female	Young	Bull	Local	SB 1176
626	Adama	Central	Male	Old	Steer	Local	SB1176
							SB0912
							SB1521
645	Adama	Central	Male	Old	Steer	Local	SB1176
							SB1522
686	Adama	Central	Male	Old	Steer	Local	SB1176
							SB1468
1264	Melge-Wondo	Central	Male	Young	Bull	Local	SB0133
2692	Hawassa	Central	Male	Young	Steer	Local	SB1176
3224	Yabello	Southeast	Female	Old	Cow	Local	SB1265
							SB1517

## Discussion

Bovine tuberculosis is an endemic disease in Ethiopia and its existence has been documented in various livestock production settings across the country. The existence of a sound disease control policy requires the availability of information based on scientific knowledge. In Ethiopia there has not been any adequate BTB and livestock movement control policy, factors that promote build-up and transmission of disease. This gave impetus to the need for investigation of genotypic characteristics and geospatial distribution of *M. bovis *strains involved in causing disease in cattle. This study investigated molecular characteristics of *M. bovis *causing gross pathologic lesions in cattle in Ethiopia. Association of specific genotypes with recorded variables such as breed, geographic origin, management system under which an animal is managed as well as forms of TB lesions dissemination in the body was also investigated. Among the significant findings of the present study were the documentation of *M. bovis *spoligotype distribution in various regions, reporting of six new spoligotypes and development of method for comparison of genetic polymorphism between the populations (sets of isolates) of *M. bovis*. The 34 strains of *M. bovis *were grouped into 12 spoligotypes (Figure 1) suggesting that TB infection in cattle in Ethiopia is caused by heterogeneous strains of *M. bovis*.

Six of the 12 (50%) spoligotypes detected in our study have not been reported from other studies. Together with six additional unique spoligotypes documented earlier [16,17], the reporting of new spoligotypes in the present study demonstrated the existence of a high degree of genetic polymorphism within the population of *M. bovis *in Ethiopia with the inherent possibility of continuing emergence of new strains over time. Likewise, several new spoligotypes have been reported recently from African countries including Uganda [18], Chad [19], Mali [20] and South Africa [21] as well as from Iran [22], showing the existence of high degree of adaptation of *M. bovis *strains to the prevailing local production settings.

The high isolation frequency of spoligotype SB1176 (41.2%) conforms to the findings of previous two works from Ethiopia [16,17] and demonstrates dominance of this spoligotype as a major cause of BTB in Ethiopian cattle particularly in the central region where the disease is highly prevalent [3]. Apart from its uniqueness, repeated isolation of SB1176 at high frequency from Ethiopia may indicate the existence of a locally evolved clonal strain with selective genetic advances perhaps linked to virulence and transmissibility. Persistent occurrence of SB1176 could also indicate the extreme high degree of reinfection rate prevailing in the area. In humans such phenomenon is attributed to 'naïve' immune response [23] or endogenous reactivation of the primary infection [24]. High stocking density and intermingling linked to growing businesses of specialized feedlot and dairy enterprises in the central region could have created a favourable environment for persistence and continued transmission of the genotype. It is of great interest to examine the current Ethiopian *M. bovis *isolates by MLVA analysis using the set of 28 different loci, in order to explore more genetic variability within identical and between different spoligotypes.

SB0134 which has previously been isolated from Mali [20], South Africa [21] and several European countries including UK [25], France [26] and Spain [10] could have been introduced into Ethiopia along with imported dairy cows (from some African and European countries) during the advent of dairy development operations around 1947. SB0133 has also been reported recently from Ethiopia [17] and Uganda [18]. Moreover, SB1468, which was isolated from Addis Ababa and southern regions (pastoral cattle), has been reported from cattle and humans in pastoral regions of Uganda [18]. The uniqueness of these spoligotypes (SB0133 and SB1468) to the East African region, as they have not been reported elsewhere, may indicate selective adaptation of the spoligotype to pastoral ecosystem. This assertion needs to be further elucidated by molecular investigation of the spoligotype over time. In a recent study [27], an epidemiologically important clonal complex of *M. bovis *(mainly characterized by absence of spacer 30) has been noted to be dominant in the West African region.

Multiple infections by two or more spoligotypes were reported in nine cases (50%) whose geographic origin was linked to Addis Ababa and its surroundings (3 cases), central (5 cases) and southeast (1 case). Out of these, two cases (321 and 422), were found to harbour five different spoligotypes each. These were young Holstein-Friesian animals whose origin was linked to dairy farms in and around Addis Ababa area, where BTB is known to be highly prevalent (23.7% and 46% individual and herd level prevalence, respectively) [28]. Stress, linked to suboptimal dairy management, prolonged life style as well as physiological characteristics (such as year round lactation and pregnancy), of dairy animals could favour high degree of super infection with different genotypes. This contradicts the work done by Duarte et al, [29] that ascribes multiple genotype infections to beef fattening practice. It is also to be noticed that multiple genotype infections have been reported in low BTB prevalence countries such as Ireland [30] and Portugal [29]. Whether or not the occurrence of multiple spoligotypes was due to exogenous super infection or reactivation of latent infection could not be ascertained at this stage, and hence needs to be further investigated by combining epidemiological information with various molecular typing methods. However, in the present study it is assumed that different sources of infection linked to intermingling of stock could be taken as a possible explanation for the reported large proportion of cattle infected with multiple genotypes. Alternatively, it could also be attributed to persistence and evolution of 'ancestral' *M. bovis *strains circulating within the country's cattle population [31]. The possibilities of new strains being acquired from other potential sources such as small ruminants and wildlife could not be ruled out as well. Under Ethiopian context, this appears plausible as inter-species co-mingling and frequent contact with wildlife is part of the traditional animal husbandry system. The reporting of identical spoligotypes from various regions across the country indicates an ongoing transmission of infection due to mainly livestock trade links between these regions.

The SEI has been calculated from previously published papers studying the *M. bovis *populations of other African countries, Iran and Canada. Comparing the spoligotype evolutionary index of the current set of Ethiopia's *M. bovis *isolates (SEI = 3.2) to the index of sets of isolates from Uganda (1.8) [18], Mali (0.56) [20], Chad (0.7) [19], Iran (0.49) [22] and Canada (0.56) [32], revealed a relatively high degree of spoligotype evolution among *M. bovis *population from Ethiopia. It appears that the spoligotypes obtained from East African region (Ethiopia and Uganda) were more polymorphic in DR region compared to isolates considered above. On the other hand the spoligotype evolution was found to be lower in the Central and West African region, which could be attributable to limited cattle imports and fairly recent introduction of the disease [19,33].

In the present study, we were not able to isolate *M. tuberculosis *from cattle and this seems to contradict with the study done by Berg et al, [17], which reported isolation of nearly 6% of *M. tuberculosis *from cattle in Ethiopia. Lack of isolation of *M. tuberculosis *from the large number of pathologic tissue samples in an environment with substantial human-animal interaction and where human tuberculosis is endemic in the local community may support the expectation that *M. tuberculosis *exhibits low virulence in cattle. This is further corroborated by the fact that *M. tuberculosis*-infected cattle often produce a rapidly vanishing infection rather than progressive disease [34,35]. Similar results were reported from Iran, where no *M. tuberculosis *was detected among 132 isolates obtained from cattle as all were confirmed to be *M. bovis *[22].

## Conclusions

In conclusion, this study provided further molecular evidence for widespread distribution of mycobacterial infection due to *M. bovis *in Ethiopian cattle population. The occurrence of multiple infections by two or more spoligotypes could indicate the existence of high degree of super infection in the area. SB1176, which has only been reported from Ethiopia, was the most dominant spoligotype. We recommend further molecular investigation of this spoligotype over time as to whether or not it represents an epidemiologically important clonal complex of *M. bovis *in the area. Compared to *M. bovis *isolates from other countries; the current isolates seem to have a relatively high degree of DR region polymorphism. The need to further elucidate epidemiological and molecular genetic characteristics of *M. bovis *strains causing infections in other domestic animals, wildlife and humans is emphasized as this may help to establish reservoirs and pattern of transmission among various hosts.

## List of abbreviations

BTB: bovine tuberculosis; CTAB: Cetyl-trimethyl-ammonium bromide; DR: direct repeat; ECL: Enhancedchemiluminescence; EHNRI: Ethiopian health and nutrition research institute; HGDI: Hunter-Gaston diversity index; MLVA: multilocus variable number of tandem repeat analysis; MoA: ministry of agriculture; MTC: *Mycobacterium tuberculosis *complex; NIPH: Norwegian institute of public health; OIE: Office international des epizooties; RFPL: restriction fragment length polymorphism; SEI: spoligotype evolutionary index; UPGMA: unweighted-pair group method with Arithmetic mean; VI: veterinary institute.

## Authors' contributions

DB: Principal investigator participated in conception and design of the study, conducted field and lab work, data analysis, drafted manuscript. ES, BD: conceived and designed the experiment, general supervision of the research work, acquisition of study fund, general conduct of the study, critical revision of the manuscript for important intellectual content. UD: participated in molecular work, data analysis, provided lab facilities and reagents, critical revision of the manuscript for important intellectual content. AB: participated in design of the study, supervision of field work, critical revision of the manuscript. JO, FA, JB: critical revision of the manuscript for important scientific content. All authors have read and approved the final version of the manuscript.

## References

[B1] Nin Pratt A Jabbar M Paulos Z Mulugeta E Dairy policies and development in South Asia and East Africa: Ethiopia case study 2005 International Livestock Research Institute (ILRI), Nairobi, Kenya

[B2] FAO Agriculture towards 2010 Proceedings of the Food and Agricultural Organization, United Nations, Conference Report C/93/24: Rome, Italy 1993

[B3] Demelash B Inangolet F Oloya J Asseged B Badaso M Yilkal A Skjerve E Prevalence of Bovine tuberculosis in Ethiopian slaughter cattle based on post-mortem examination Trop Anim Health Prod 2009 41 5 755 65 10.1007/s11250-008-9248-9 19058024

[B4] Hardie RM Watson JM *Mycobacterium bovis *in England and Wales: past, present and future Epidemiol Infect 1992 109 1 23 33 1499671 PMC2272235

[B5] Daborn CJ Grange JM Kazwala RR The bovine tuberculosis cycle-an African perspective Soc Appl Bacteriol Symp Ser 1996 25 27S 32S 8972117 10.1111/j.1365-2672.1996.tb04595.x

[B6] Ayele WY Neill SD Zinsstag J Weiss MG Pavlik I Bovine tuberculosis: an old disease but a new threat to Africa Int J Tuberc Lung Dis 2004 8 8 924 37 15305473

[B7] Achtman M Sussmann M A phylogenetic perspective on molecular epidemiology Molecular Medical Microbiology 2001 San Diego: Academic Press 485 59

[B8] Dvorska L Bartos M Martin G Erler W Pavlik I Strategies for differentiation, identification and typing of medically important species of mycobacteria by molecular methods Vet Med-Czech 2001 46 11-12 309 328

[B9] Rodriguez S Romero B Bezos J de Juan L Alvarez J Castellanos E Moya N Lozano F Gonzalez S Saez-Llorente JL Mateos A Dominguez L Aranaz A High spoligotype diversity within a *Mycobacterium bovis *population: Clues to understanding the demography of the pathogen in Europe Vet Microbiol 2010 141 89 95 10.1016/j.vetmic.2009.08.007 19720476

[B10] Aranaz A Liebana E Mateos A Dominguez L Vidal D Domingo M Gonzolez O Rodriguez-Ferri EF Bunschoten AE Van Embden JD Cousins D Spacer oligonucleotide typing of *Mycobacterium bovis *strains from cattle and other animals: a tool for studying epidemiology of tuberculosis J Clin Microbiol 1996 34 11 2734 40 8897175 10.1128/jcm.34.11.2734-2740.1996 PMC229396

[B11] Haddad N Masselot M Durand B Molecular differentiation of *Mycobacterium bovis *isolates. Review of main techniques and applications Res Vet Sci 2004 76 1 1 18 10.1016/S0034-5288(03)00078-X 14659724

[B12] OIE Bovine tuberculosis Manual of diagnostic tests and vaccines for terrestrial animals (mammals, birds and bees) 2009 Chapter 2.4.7 683

[B13] Pace JE Waleman DL Determining the age of cattle by their teeth 2003 Department of Animal Science, Institute of Food and Agriculture (IFAS), Florida Cooperative Extension Service, University of Florida, USA

[B14] Dale JW Brittain D Cataldi AA Cousins D Crawford JT Driscoll J Heersma H Lillebaek T Quitugua T Rastogi N Skuce RA Sola C Van Soolingen D Vincent V Spacer oligonucleotide typing of bacteria of the *Mycobacterium tuberculosis complex*: recommendations for standardised nomenclature Int J Tuberc Lung Dis 2001 5 3 216 9 11326819

[B15] Hunter PR Gaston MA Numerical index of the discriminatory ability of typing systems: an application of Simpson's index of diversity J Clin Microbiol 1988 26 11 2465 2466 3069867 10.1128/jcm.26.11.2465-2466.1988 PMC266921

[B16] Ameni G Aseffa A Sirak A Engers H Young DB Hewinson RG Vordermeier MH Gordon SV Effect of skin testing and segregation on the prevalence of bovine tuberculosis, and molecular typing of *Mycobacterium bovis*, in Ethiopia Vet Rec 2007 161 23 782 6 18065813 PMC2292248

[B17] Berg S Firdessa R Habtamu M Gadisa E Mengistu A Yamuah L Ameni G Vordermeier M Robertson BD Smith NH Engers H Young D Hewinson RG Aseffa A Gordon SV The burden of mycobacterial disease in Ethiopian cattle: implications for public health PLoS One 2009 4 4 e5068 10.1371/journal.pone.0005068 19352493 PMC2662418

[B18] Oloya J Kazwala R Lund A Opuda-Asibo J Demelash B Skjerve E Johansen TB Djønne B Characterisation of mycobacteria isolated from slaughter cattle in pastoral regions of Uganda BMC Microbiol 2007 7 95 10.1186/1471-2180-7-95 17961243 PMC2140064

[B19] Diguimbaye-Djaibe C Hilty M Ngandolo R Mahamat HH Pfyffer GE Baggi F Hewinson G Tanner M Zinsstag J Schelling E *Mycobacterium bovis *isolates from tuberculous lesions in Chadian zebu carcasses Emerg Infect Dis 2006 12 5 769 71 16704835 10.3201/eid1205.050691 PMC3374448

[B20] Muller B Steiner B Bonfoh B Fane A Smith NH Zinsstag J Molecular characterisation of *Mycobacterium bovis *isolated from cattle slaughtered at the Bamako abattoir in Mali BMC Vet Res 2008 4 26 10.1186/1746-6148-4-26 18637160 PMC2483712

[B21] Michel AL Hlokwe TM Coetzee ML Mare L Connoway L Rutten VP Kremer K High *Mycobacterium bovis *genetic diversity in a low prevalence setting Vet Microbiol 2008 126 1-3 151 9 10.1016/j.vetmic.2007.07.015 17720336

[B22] Tadayon K Mosavari N Sadeghi F Forbes KJ *Mycobacterium bovis *infection in Holstein Friesian cattle, Iran Emerg Infect Dis 2008 14 12 1919 21 10.3201/eid1412.070727 19046521 PMC2634609

[B23] Bates JH Stead WW Rado TA Phage type of tubercle bacilli isolated from patients with two or more sites of organ involvement Am Rev Respir Dis 1976 114 2 353 358 823847 10.1164/arrd.1976.114.2.353

[B24] du Plessis DG Warren R Richardson M Joubert JJ van Helden PD Demonstration of reinfection and reactivation in HIV-negative autopsied cases of secondary tuberculosis: multilesional genotyping of *Mycobacterium tuberculosis *utilizing IS 6110 and other repetitive element-based DNA fingerprinting Tuberculosis 2001 81 3 211 220 10.1054/tube.2000.0278 11466033

[B25] Gibson AL Hewinson G Goodchild T Watt B Story A Inwald J Drobniewski FA Molecular epidemiology of disease due to *Mycobacterium bovis *in humans in the United Kingdom J Clin Microbiol 2004 42 1 431 4 10.1128/JCM.42.1.431-434.2004 14715798 PMC321667

[B26] Haddad N Ostyn A Karoui C Masselot M Thorel MF Hughes SL Inwald J Hewinson RG Durand B Spoligotype diversity of *Mycobacterium bovis *strains isolated in France from 1979 to 2000 J Clin Microbiol 2001 39 10 3623 32 10.1128/JCM.39.10.3623-3632.2001 11574583 PMC88399

[B27] Muller B Hilty M Berg S Garcia-Pelayo MC Dale J Boschiroli ML Cadmus S Ngandolo BN Godreuil S Diguimbaye-Djaibe C Kazwala R Bonfoh B Njanpop-Lafourcade BM Sahraoui N Guetarni D Aseffa A Mekonnen MH Razanamparany VR Ramarokoto H Djønne B Oloya J Machado A Mucavele C Skjerve E Portaels F Rigouts L Michel A Muller A Kallenius G van Helden PD Hewinson RG Zinsstag J Gordon SV Smith NH African 1, an epidemiologically important clonal complex of *Mycobacterium bovis *dominant in Mali, Nigeria, Cameroon, and Chad J Bacteriol 2009 191 6 1951 1960 10.1128/JB.01590-08 19136597 PMC2648362

[B28] Elias K Hussein D Asseged B Wondwossen T Gebeyehu M Status of bovine tuberculosis in Addis Ababa dairy farms Rev Sci Tech 2008 27 3 915 923 19284060 10.20506/rst.27.3.1850

[B29] Duarte EL Domingos M Amado A Botelho A Spoligotype diversity of *Mycobacterium bovis *and *Mycobacterium caprae *animal isolates Vet Microbiol 2008 130 3-4 415 21 10.1016/j.vetmic.2008.02.012 18417301

[B30] Costello E O'Grady D Flynn O O'Brien R Rogers M Quigley F Egan J Griffin J Study of restriction fragment length polymorphism analysis and spoligotyping for epidemiological investigation of *Mycobacterium bovis *infection J Clin Microbiol 1999 37 10 3217 22 10488180 10.1128/jcm.37.10.3217-3222.1999 PMC85531

[B31] Milian-Suazo F Banda-Ruiz V Ramirez-Casillas C Arriaga-Diaz C Genotyping of *Mycobacterium bovis *by geographic location within Mexico Prev Vet Med 2002 55 4 255 64 10.1016/S0167-5877(02)00015-6 12392876

[B32] Lutze-Wallace C Turcotte C Sabourin M Berlie-Surujballi G Barbeau Y Watchorn D Bell J Spoligotyping of *Mycobacterium bovis *isolates found in Manitoba Can J Vet Res 2005 69 2 143 5 15971679 PMC1142182

[B33] Cadmus S Palmer S Okker M Dale J Gover K Smith N Jahans K Hewinson RG Gordon SV Molecular analysis of human and bovine tubercle bacilli from a local setting in Nigeria J Clin Microbiol 2006 44 1 29 34 10.1128/JCM.44.1.29-34.2006 16390943 PMC1351927

[B34] Krishnaswami KV Mani KR *Mycobacterium tuberculosis humanis *causing zoonotic tuberculosis among cattle Indian J Public Health 1983 27 2 60 3 6668064

[B35] Cousins DV Huchzermeyer HFK Griffin JFT Bruckner GK Van rensburg IBJ kriek NPJ Coetzer AW, Tustin RC Tuberculosis Infectious Diseases of Livestock 2004 3 2 Cape Town: Oxford University Press 1973 1993

